# An Open Source Automated Bar Test for Measuring Catalepsy in Rats

**DOI:** 10.1523/ENEURO.0488-19.2020

**Published:** 2020-06-19

**Authors:** Karling R. Luciani, Jude A. Frie, Jibran Y. Khokhar

**Affiliations:** Department of Biomedical Sciences, Ontario Veterinary College, University of Guelph, Guelph, Ontario, N1G2W1, Canada

**Keywords:** antipsychotic, automated, catalepsy, open source

## Abstract

Catalepsy bar tests are widely used to measure the failure to correct an imposed posture resulting from muscular rigidity. Procedures for measuring catalepsy vary greatly in the published literature, but one commonly used test measures the time it takes for a rodent to remove one or both of its forelimbs from a bar. The following paper describes an affordable, adjustable, open-source bar test that automatically measures and logs the time it takes for a rat to remove itself from a bar. While commercially available automated bar tests are prohibitively expensive, requiring proprietary software and hardware to operate, the proposed apparatus runs on an Arduino-based microcontroller making it low-cost and customizable. This 3D-printed design costs less than 65 United States dollars to build and is simple to assemble and operate. The beam-break sensor design also eliminates many of the pitfalls of the “complete-the-circuit”-based approach to recording catalepsy. The paper further describes the successful validation of the design using adult male rats injected with different doses of haloperidol to demonstrate a dose-dependent cataleptic effect. This design provides a versatile, low-cost solution to standardizing and automating measurement of catalepsy in rodents.

## Visual Abstract

**Figure:**
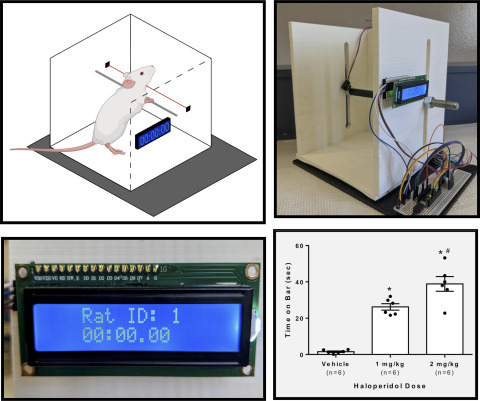

## Significance Statement

Catalepsy measurements are important in behavioral research involving antipsychotic drugs, Parkinson’s disease, schizophrenia, and cannabis exposure. Standardized ways of measuring catalepsy will lead to more accurate data, while allowing for consistency between studies. To this end, we designed and constructed a catalepsy bar test that automatically measures and records the time it takes for a rat to remove its forelimbs from a bar. This easy-to-build apparatus will allow for unbiased, human-error free measurements to be made at a very low cost.

## Introduction

Catalepsy is a behavioral state of muscular rigidity that is associated with antipsychotic drugs (Banasikowski and Beninger, 2012), type-1 cannabinoid (CB1) agonists (Metna-Laurent et al., 2017), and several other drug classes (Alvarez-Cervera et al., 2005). Catalepsy also has similarities to symptoms in Parkinson’s disease (Alam and Schmidt, 2002) and catatonic schizophrenia (Sanberg et al., 1988). Catalepsy can be observed in rodents when they are placed in an abnormal position and remain in this position for a prolonged period of time (Banasikowski and Beninger, 2012). Studies have shown that catalepsy is related to decreased dopamine (DA) transmission at postsynaptic D2 receptors (Wadenberg et al., 2000). Catalepsy can also result from central CB1 receptor agonism, and is therefore an important part of the behavioral tetrad test for cannabinoid-like pharmacological agents (Metna-Laurent et al., 2017).

Current ways of measuring catalepsy intensity in rodents is influenced by minor methodological differences, and thus have not produced consistent results across laboratories (Sanberg et al., 1988). The typical test for measuring catalepsy is the bar test which consists of placing a rat’s forepaws on an elevated bar with the hind paws remaining on the floor. The time recorded for the rat to correct this posture is an index of the intensity of catalepsy. A cataleptic rat will continue to hold onto the bar for a prolonged period of time while a normal rat will change its position within seconds (Sanberg et al., 1988). This test has numerous variations used in research. Some studies stop the timer when the rat removes a single forepaw (Sanberg et al., 1988), while others wait until both paws are removed (Bricker et al., 2014). Rats also vary in size and if a fixed bar is too high, both the cataleptic and normal rats are unlikely to rest on the bar for an appreciable length of time (Sanberg et al., 1988).

To avoid bar height and human errors or bias as factors that may influence the catalepsy measurements, we created an open source, 3D printed, automated catalepsy bar test with adjustable bar heights. The test apparatus includes an automated timer connected to beam-break sensors which are attached directly above the bar. The bar can be moved vertically to adjust the height appropriate for the animal’s size. The timer starts once the rat’s forepaws are resting on the bar. When the rat descends from this posture, the break-beam sensors cause the timer to stop and the time is shown on an LCD display. The data are logged to a .csv file on an SD card. A push button saves the elapsed time into the logging module. The addition of data logging allows the researcher more potential for performing the behavioral measure without additional help. Haloperidol is a D2 receptor antagonist that leads to the development of catalepsy in rats (Banasikowski and Beninger, 2012). The apparatus is validated by injecting rats with a 1 or 2 mg/kg dose of haloperidol to ensure the rats are exhibiting a cataleptic response compared with vehicle measurements.

## Materials and Methods

### Build instructions

#### 3D print housing

Download the “Catalepsy_stage.STL” file from https://www.khokharlab.com/open-source-file-downloads. The housing should be printed upright. No structural support should be needed. The printer filament used for this design was polyactic acid; however, any filament should work equally well. If the 3D printer being used is too small to print the housing in one piece, the housing can be sliced, printed in several pieces, and epoxied together.

#### Circuit assembly

**Figure 1.**
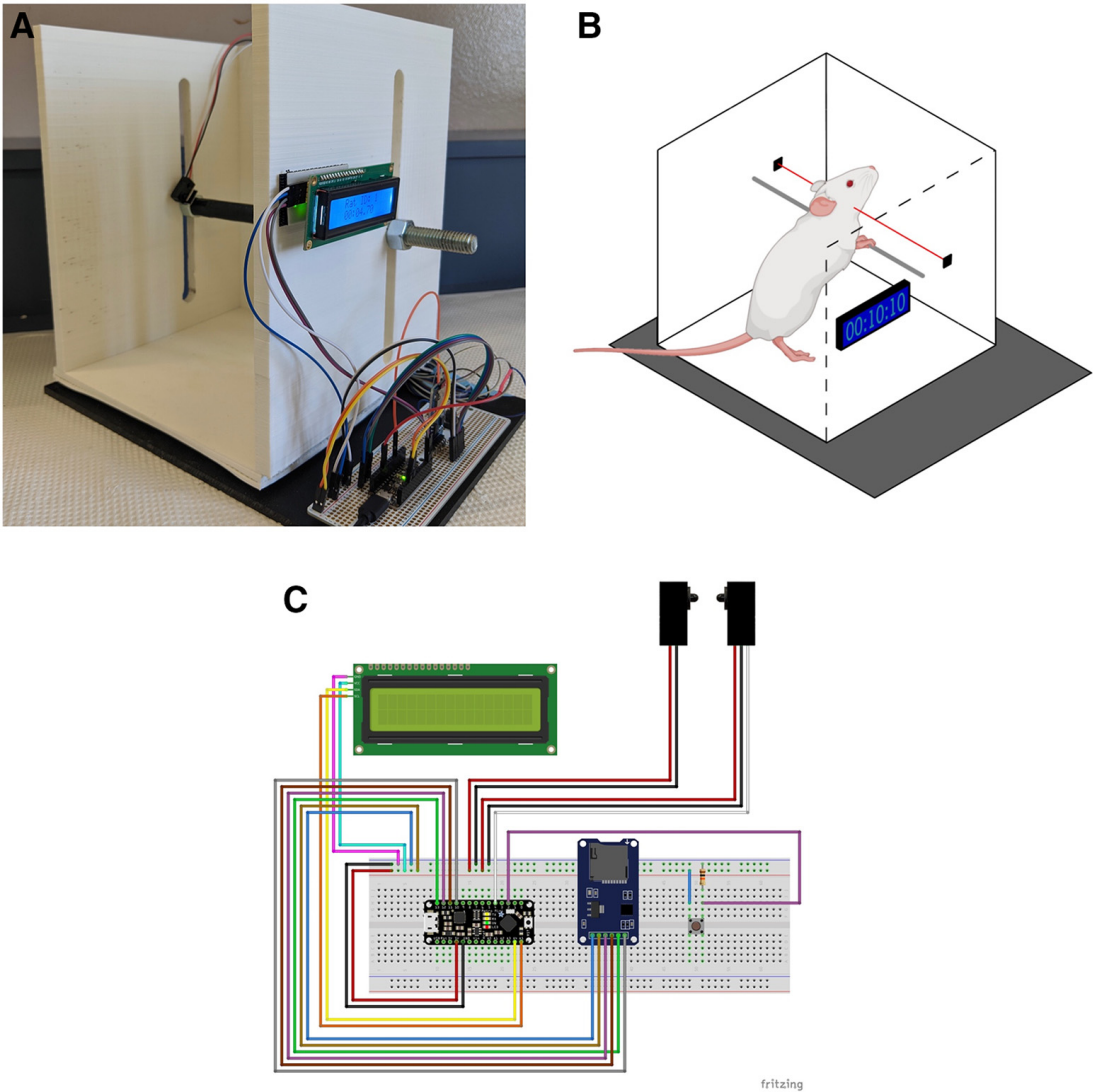
3D printed, automated catalepsy bar test (***A***), diagram of a rat in the bar test (***B***), and circuit schematic for automated bar test assembly (***C***).

Assemble the circuit following the circuit schematic seen in Figure 1*C* using the parts listed in Table 1.

**Table 1:** Bill of materials

Quantity	Component name	Website	Price (United States)
1	Adafruit Metro Mini 328	https://www.adafruit.com/product/2590	12.50
1	Keyestudio 1602 LCD IIC/I2C/TWI Display 16x2	https://www.amazon.com/Keyestudio-Display-Module-Arduino-raspberry/dp/B01799UUGS/	7.99
1	IR Break Beam Sensor - 3mm	https://www.adafruit.com/product/2167	1.95
1	5V MicroUSB Power Supply	https://www.amazon.com/Travel-Charger-Adapter-Samsung-Galaxy/dp/B0117O020U/ref=sr_1_3?keywords=5v+micro+usb+power+supply&qid=1580226650&sr=8-3	6.79
1	Adafruit Perma-Proto Breadboard	https://www.adafruit.com/product/1606	6.95
1	Jumper Wires	https://www.adafruit.com/product/1957	1.95
1	Threaded Steel Rod - 0.5” diameter, 12” length	https://www.homedepot.ca/product/paulin-1-2-13x12-inch-thread-rod-plated-/1000797593	1.64
1	10 kΩ resistor	https://www.adafruit.com/product/2784	0.75
1	MicroSD Card Adaptor	https://www.amazon.com/HiLetgo-Adater-Interface-Conversion-Arduino/dp/B07BJ2P6X6/ref=sr_1_2?keywords=sd+card+arduino&qid=1580155120&s=electronics&sr=1-2	6.99
4	0.5” Nuts	https://www.homedepot.ca/product/paulin-1-2-13-fin-hex-nuts-gr2-unc/1000123145	1.30
1	Push Button	https://www.adafruit.com/product/1119	2.50
1	PLA Filament (2/3 roll)	https://www.amazon.com/Printer-Filament-SUNLU-Dimensional-Accuracy/dp/B07XG3RM58/ref=sr_1_6?keywords=pla+filament&qid=1580155473&sr=8-6	17.99(2/3 = 11.99)
1	Additional wire	https://www.homedepot.ca/product/southwire-simpull-t90-copper-electrical-wire-14-solid-blue-300m/1000150577	0.42

Total cost = $63.72.

#### Assemble bar

Place bar through apparatus and attach nuts so as to “sandwich” the walls between the nuts. Heat shrink or electrical tape can be placed on the bar to make it more comfortable for the rat. Glue sensors to inside nuts and face them toward each other.

#### Firmware

Download and install the Arduino IDE from https://www.arduino.cc/en/main/software. Open the Arduino IDE and navigate to Sketch/Include Library/SD. Download LiquidCrystal_I2C.h from https://drive.google.com/file/d/14VPCVHqlfbtxAn5LCF3tGZytkGEMrk3Q/view and place it in the Arduino Libraries folder. Download the “Counter.ino” program from https://www.khokharlab.com/open-source-file-downloads. Flash code to Arduino. The Arduino IDE is compatible with all major operating systems.

### Operating instructions

The LCD display will count in 1/100th seconds when the IR beam is broken. When the beam resumes, the count will stop. After a period of 3 s, the beam can be broken again to restart the time, or the push button can be pressed to save the current value. This 3-s delay allows for the rat to be removed from the apparatus without the experimenter accidentally clearing the count. Adjust the height of the bar to an appropriate height for the size of the rat. Place the rat’s forepaws on the bar quickly as the count will start immediately on the beam being broken. After the rat’s removal of itself from the imposed posture, the LCD will then display the current rat ID and the time in min:s.hundredths (00:00.00) format. Press the push button to add the recorded time to the data module.

### Code accessibility

The code/software described in the paper is freely available online at https://www.arduino.cc/en/main/software. The code is also available as Extended Data Figure 2-1.

**Figure 2.**
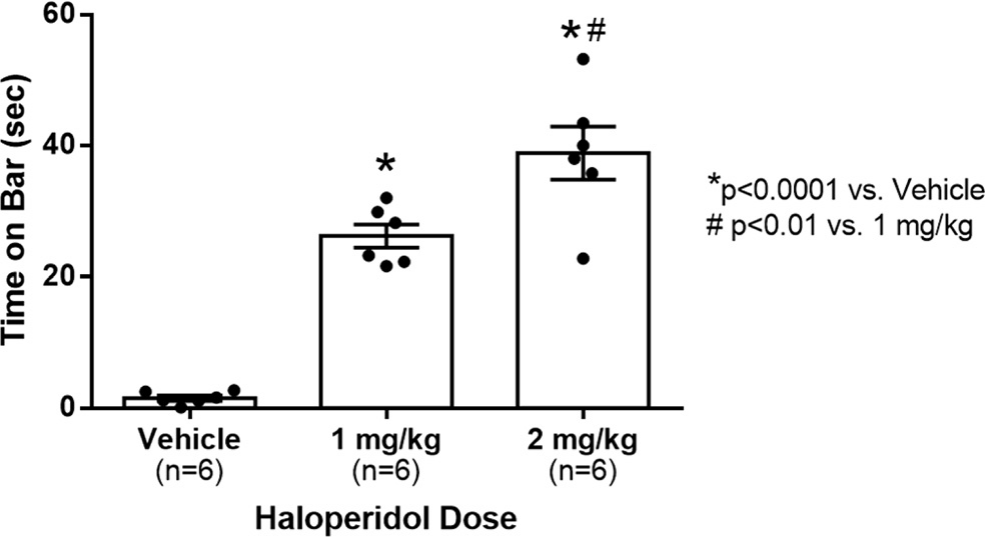
Mean time spent on catalepsy bar in seconds as measured by the apparatus in adult male rats. Error bars represent SEM; *p < 0.0001 compared with vehicle; #*p* < 0.01 compared with 1 mg/kg dose. Raw data included as Extended Data Figure 2-1.

10.1523/ENEURO.0488-19.2020.f2-1 Extended Data Figure 2-1 A sample file from the logger with the raw data used for Figure 2. Download Figure 2-1, CSV file

### Validation

#### Animals

Male Sprague Dawley rats (*n* = 18) ordered from Charles River were kept in ventilated polyethylene cages on a 12/12 h light/dark cycle at room temperature with *ad libitum* food (14% TekLad rat chow) and water. Rats weighing approximately 400 g were pair housed. All animal procedures were performed in accordance with the University of Guelph animal care committee’s regulations.

#### Drug

Haloperidol was dissolved in 0.5 N acetic acid. The drug was then brought to pH 5.5. Injections were administered intraperitoneally at a dose of 1 or 2 mg/kg free base and were compared with a saline vehicle. This dose was chosen as it has been shown to robustly produce catalepsy (Costall et al., 1978; Bricker et al., 2014).

#### Catalepsy bar test

The bar test was set to a height of 12 cm. Rats were gently positioned, placing their forelimbs on the bar and their hindlimbs on the floor of the apparatus. A researcher measured the time for the rat to remove both paws from the bar using a stopwatch for comparison with the automated measurements. Baseline measurements were taken before injection with haloperidol. Haloperidol induced catalepsy was measured 1 h after intraperitoneal injection.

#### Statistical analysis

Statistical analyses were performed using SPSS. Figures were made using GraphPad Prism. A one-way ANOVA was used to compare time on the bar after haloperidol or saline treatment followed by *post hoc* comparisons with Bonferroni corrections.

## Results

The results of the experiment are shown in Figure 2. One-way ANOVA revealed a significant effect of dose (*F*_(2,18)_ = 54.345, *p *<* *0.0001, η^2^ = 0.879). Rats given either dose showed a significantly increased amount of time on the bar (*p *<* *0.0001) compared with vehicle, and the 2 mg/kg dose spent significantly more time on the bar than the 1 mg/kg dose (*p *<* *0.01).

## Discussion

The influence of minor methodological differences in catalepsy measurements have been shown to cause large differences in results leading researchers to desire automated testing (Sanberg et al., 1988). The results from this experiment verify the proposed automated catalepsy bar test as a versatile automated measuring apparatus that is able to standardize measurements of catalepsy in rats. The apparatus was used to observe a robust dose-dependent effect of haloperidol on catalepsy in rats (Fig. 1), as has been seen in previous studies (Sanberg et al., 1988). In addition, this automated apparatus makes it possible for a single experimenter to test catalepsy in animals without help with the addition of the data logging module; this apparatus helps reduce the possibility of observer bias as the sensors are blind to the treatment status of the animals.

Existing commercial automated bar tests such as the catalepsy test chamber made by Med Associates use a “complete the circuit” approach that measures the time that contact is maintained between the bar and the floor as a complete electrical circuit. One study using the catalepsy test chamber found problems with rats unable to grip the bar properly as a result of catalepsy resulting in electrical contact not being made, and thus recording incorrect measurements (Bricker et al., 2014). This problem is not seen in our apparatus as measurements are based on the location of the rat rather than the contact being made.

Another open-source automated test exists that uses the same complete the circuit method mentioned previously (Alvarez-Cervera et al., 2005). While the authors attempted to fix the gripping issue by using a very thin diameter bar (0.4 cm), the problem can still arise when a rat adjusts itself such that another part of their forelimbs are making contact with the bar. Any portion of the forelimbs other than the paws are covered in fur and will fail to make electrical contact. The design also used wood that made sterilization difficult and still cost an estimated $500 US to build. The present apparatus’ housing is made of polylactic acid (PLA) which is easy to sterilize and costs under 65 United States dollars to build. The adjustable bar height also allows for this apparatus to be used with rats of different ages and sexes, and has been successfully used by our group with mice as well.

However, our design is not without its limitations. While our detection method does measure the intensity of catalepsy, it does not capture postural shifts or cataleptic movements of the limbs. One potential method that we can foresee capturing those elements in future designs would be adding force or load sensors to the incline plane version of the catalepsy task, which would allow us to measure postural shifts as the angle is increased until the rat falls off the plane. Overall, the automated catalepsy bar test proposed in this paper provides standardized testing of catalepsy in rats with catalepsy intensity logged automatically for improved efficiency over manual measurements. The apparatus is easy to build, inexpensive, and accessible for other researchers to create and modify to fit their needs.

## Synthesis

Reviewing Editor: Alexxai Kravitz, Washington University in St. Louis Decisions are customarily a result of the Reviewing Editor and the peer reviewers coming together and discussing their recommendations until a consensus is reached. When revisions are invited, a fact-based synthesis statement explaining their decision and outlining what is needed to prepare a revision will be listed below. The following reviewer(s) agreed to reveal their identity: NONE.

This manuscript was accepted after editorial review.
